# Why Patient-Reported Outcome Measures Have Not Reached Primary Eye Care: A Critical Narrative Review of the Targeting, Access, and Action Gaps

**DOI:** 10.3390/jcm15218461

**Published:** 2026-10-30

**Authors:** Jamolbi Rahmatova, Viraj Shah, Shahnia Surendran, Arian Arjomandi Rad, Robert Vardanyan

**Affiliations:** 1 University College London Hospitals NHS Foundation Trust, London NW1 2BU, UK; 2 Imperial College Healthcare NHS Trust, London W2 1NY, UK; 3 Department of Cardiothoracic Surgery, Oxford University Hospitals NHS Foundation Trust, Oxford OX3 9DU, UK; 4 Department of Neurosurgery, University Hospitals Plymouth NHS Foundation Trust, Plymouth PL6 8DH, UK; robert.vardanyan1@nhs.net

**Keywords:** patient-reported outcome measures, primary eye care, optometry, visual function, Rasch analysis, implementation science, measurement targeting, health equity

## Abstract

Eye care is shifting into community and primary care settings, yet patient-reported outcome measures (PROMs) have not followed. Non-adoption is routinely attributed to clinician time, information technology, and absent guidance, explanations that are real but have not changed practice. This critical narrative review synthesises evidence across ophthalmic PROM psychometrics, implementation science, community eye care service evaluations, and primary eye care epidemiology, with every primary source verified against its PubMed record. We propose, as hypotheses requiring validation rather than as established findings, four structural gaps: instruments calibrated in hospital and surgical cohorts may mistarget milder community populations; in the only published implementation reporting uptake, patients with lower visual acuity were less likely to attempt a self-administered vision PROM, suggesting that non-completion may be informative rather than random; we identified no primary eye care study in which scores were returned to the consultation, the step at which benefit has been demonstrated in other specialties; and the high-volume community case mix remains largely unmeasured. Beneath these, we propose a fifth, cross-cutting funding gap, since we identified no routine reimbursement route for this activity, although the service and financing evidence available to us is predominantly from the United Kingdom. We set out a staged implementation route, the observations that would confirm or refute each proposed gap, and the strongest counter-argument to our own position, namely that non-adoption may be rational where instruments carry little decision-relevant information in mild disease.

## 1. Introduction

Globally, an estimated 43.3 million people were blind in 2020, with a further 295 million experiencing moderate or severe distance vision impairment and 510 million living with vision impairment from uncorrected presbyopia [1]. Much of this burden is avoidable, and much of it is managed, or ought to be managed, outside hospitals. Health systems in the United Kingdom, Australia, Canada, and elsewhere have accordingly moved substantial volumes of eye care into community optometry, shared-care schemes, and primary care-led services.

Patient-reported outcome measures are standardised instruments completed by patients to quantify symptoms, functioning, and health-related quality of life from their own perspective. In ophthalmology, they capture what visual acuity and intraocular pressure cannot: difficulty with night driving, glare, dependence on others, and the emotional consequences of losing sight [2,3]. Their value is not seriously disputed. A Cochrane review of 116 randomised trials involving 49,785 patients found that feeding PROM data back to clinicians and patients probably improves patient–clinician communication, rates of diagnosis and notation, and disease control, with smaller gains in quality of life [4].

Yet in routine ophthalmic practice, adoption has stalled. A cross-sectional survey of ophthalmologists in the United Kingdom, Germany, and Switzerland found that only 31% of 112 respondents used PROMs at all, and use was concentrated in cataract services [5]. In primary eye care specifically, systematic collection is close to non-existent. The explanations usually offered, namely the consultation time, poorly designed information technology, and absent guidance on instrument selection, are well documented and undoubtedly contribute [6,7]. They are also, we argue, insufficient. These same barriers apply in oncology, psychiatry, and rheumatology, where PROM programmes have nevertheless been established at scale.

One finding from the European survey is particularly difficult to reconcile with the standard account: perceived usefulness did not predict adoption [5]. Clinicians who believed PROMs were valuable were no more likely to use them. If the obstacle were awareness, persuasion, or education, that relationship should hold. It does not. Something structural is intervening between belief and practice.

**Figure 1:**
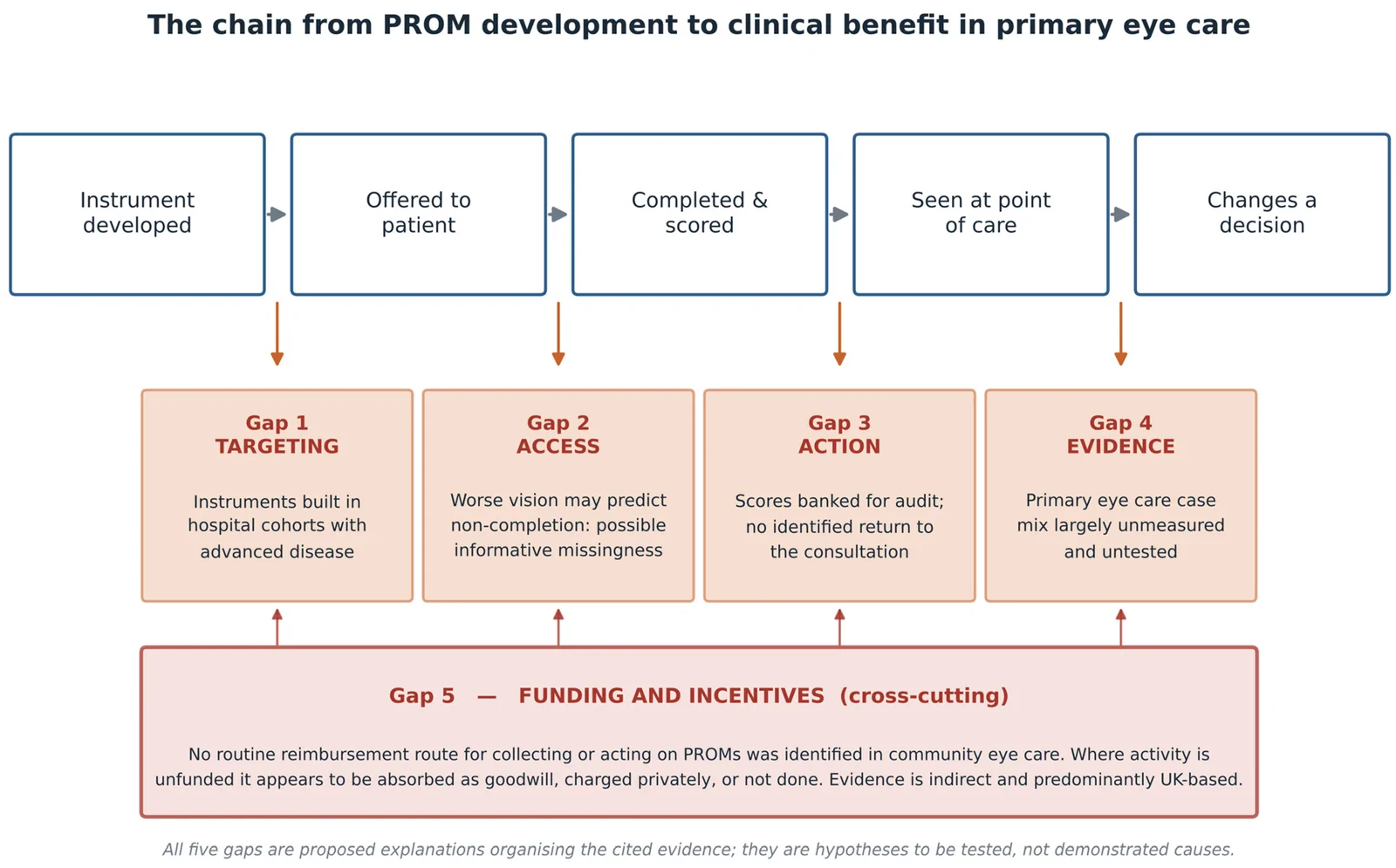
The chain from PROM development to clinical benefit in primary eye care. Four proposed gaps are shown interrupting specific links, with a fifth, funding, acting as the substrate beneath all of them. Generic implementation barriers such as clinician time and information technology operate across every link but do not account for the specific failures at each stage. The five gaps are explanations that we propose to organise the cited evidence; they are hypotheses to be tested rather than demonstrated causes.

This review advances a different explanation, which we offer as a structured hypothesis rather than a demonstrated account. We propose that PROMs have failed to reach primary eye care for reasons rooted in measurement science, service design, and payment and that four specific gaps, namely targeting, access, action, and evidence, may each independently break the chain between instrument development and clinical benefit, while a fifth, funding, appears to act across all of them (Figure 1). Throughout, we have sought to keep the strength of each claim proportional to the evidence behind it, and we flag explicitly where an argument rests on extrapolation from hospital populations, on a single study, or on evidence drawn from a single health system. We then set out the strongest case against our own argument, the observations that would distinguish the two accounts, and what would have to change, in what order, for the chain to close.

## 2. Approach to the Literature

This is a critical narrative review, not a systematic review, and it makes no claim of exhaustive retrieval. The design was adopted deliberately, because of the nature of the literature. The question that we address, namely why an intervention with demonstrated benefit elsewhere has failed to establish itself in a particular setting, requires synthesis across bodies of work that share no common indexing vocabulary and would not be captured by any single reproducible search string. A systematic search restricted to studies reporting PROM use in primary eye care returns a small and heterogeneous set of reports, most of which are descriptive, and excludes precisely the psychometric and implementation evidence needed to explain the pattern. We use “critical narrative review” throughout in the sense defined by SANRA [8]: a non-systematic synthesis that states its search and selection methods transparently, appraises the evidence it cites, and distinguishes argument from demonstration.

### 2.1. Search Strategy

We searched MEDLINE via PubMed up to June 2026, supplemented by backward and forward citation tracking from key papers and by targeted hand-searching of the reference lists of included reviews. Four domains were searched separately, because no single string spans them: (i) development and psychometric evaluation of vision-specific PROM instruments, with particular attention to Rasch analyses reporting item-to-person targeting; (ii) implementation studies and evidence syntheses concerning PROM adoption in routine care; (iii) service evaluations of community, optometry-led, and primary care-based eye services; and (iv) epidemiological data describing the primary eye care case mix. The principal concepts combined in each domain were, respectively, patient-reported outcome measures with ophthalmic and psychometric terms; patient-reported outcome measures with implementation, barrier, and facilitator terms restricted to reviews; optometry and primary eye care service terms with evaluation, referral, and commissioning terms; and ophthalmic disease terms with prevalence and population terms. The full search strings are given in Appendix A.

### 2.2. Study Selection

Titles and abstracts were screened against the purpose of each domain rather than against formal eligibility criteria. For domain (i), we sought primary psychometric evaluations of named vision PROMs, prioritising those reporting person and item locations on a common scale; for domain (ii), we sought systematic reviews, umbrella reviews, and implementation studies reporting barriers, facilitators, or the effects of PROM feedback; for domain (iii), we sought evaluations of services actually delivering eye care outside the hospital, with reported activity or outcome data; and for domain (iv), we sought population-level estimates of the conditions presenting to such services. Records were retained when they contributed evidence bearing on one of the five proposed gaps, and systematic reviews, population-based studies, and primary psychometric analyses were preferred over narrative accounts and single-case reports. Thirty-three sources are cited.

Selection was iterative and purposive rather than protocol-driven, and no review protocol was registered, consistent with the narrative design set out in Section 2. The citation set should accordingly be read as evidence assembled in support of an argument and appraised as such, rather than as the complete retrievable evidence base on this question. The search strings in Appendix A are reported in full so that the yield can be reproduced and independently screened.

### 2.3. Distinguishing Evidence from Inference

Because a central risk in work of this kind is that the argument and evidence become difficult to separate, we have adopted a consistent convention. Where a statement describes what a cited study directly observed, it is written as a finding and attributed to that study. Where a statement extends such a finding to a population, setting, or health system that the study did not examine, it is identified as an inference, an extrapolation, or a hypothesis, and the direction and basis of the extrapolation are stated. Table 2 records, for each proposed gap, the strength and provenance of the supporting evidence alongside the proposed response, and Section 10 sets out the specific points at which our argument depends on extrapolation rather than observation.

### 2.4. Verification, Reporting Standard, and Patient Involvement

Citation verification. Every primary source cited in this review was checked against its PubMed record for authorship, journal, year, volume, and pagination before inclusion. We report this explicitly because reference inaccuracy is a growing and under-acknowledged problem in the review literature and because a review of this kind depends entirely on the accuracy of its sources. Assertions that could not be traced to a verifiable indexed record were removed rather than retained with approximate attribution.

Reporting standard. This review was prepared with reference to SANRA, the Scale for the Assessment of Narrative Review Articles, which addresses the justification of importance and aims, a description of the literature search, referencing, a presentation of the evidence level, and a presentation of relevant endpoint data [8].

Patient and public involvement. No patients or members of the public were involved in the conception, design, or drafting of this review. We consider this a substantive limitation given that our central argument concerns the mismatch between instruments and the patients asked to complete them, and we recommend that any programme following the route proposed in Section 9 be co-designed with patients from the outset.

Readers should weigh the findings accordingly. A purposive synthesis carries a real risk of selective citation, and we return to this in the limitations.

## 3. Defining Primary Eye Care and Who It Sees

Any argument about measurement must begin with the population being measured and with a clear definition of the setting. Terminology here is not standardised across health systems, and a reviewer from one country may not recognise the arrangements of another.

Definition. By primary eye care, we mean the first point of professional contact for an eye problem, delivered outside hospital ophthalmology and without prior specialist referral. In the United Kingdom, this is predominantly community optometry, operating through General Ophthalmic Services sight testing and locally commissioned enhanced schemes such as Minor Eye Conditions Services; in Australia and Canada, independent optometric practice fulfils a comparable role; in the United States, optometry and primary care physicians share it; and in many low- and middle-income settings, the equivalent function sits with primary health facilities, ophthalmic clinical officers, and community outreach. What unites these arrangements is not the professional group or funding model but position in the pathway: undifferentiated presentations, unselected severity, and responsibility for deciding who needs onward referral.

### 3.1. The Observed Case Mix

Service evaluation data give a reasonably concrete picture. In a twelve-month evaluation of the Lambeth and Lewisham Minor Eye Conditions Scheme, 2123 patients accessed the service; the most common presenting reasons were red eye (36.7%), painful white eye (11.1%), and flashes and floaters (10.2%). Community optometrists managed 64.1% of patients without referral, and 18.9% were referred to hospital eye services, of which 89.2% were judged appropriate. First hospital attendances referred by general practitioners fell by 26.8% (95% CI −40.5% to −13.1%) relative to a neighbouring area without the scheme [9].

Alongside this acute workload sits a large burden of chronic and refractive need. Dry eye disease has a reported prevalence of 5–50% depending on the definition applied, with symptoms and signs diverging substantially [10]. Uncorrected presbyopia affects an estimated 510 million people worldwide, and a further 295 million experience moderate or severe distance vision impairment [1].

**Figure 2:**
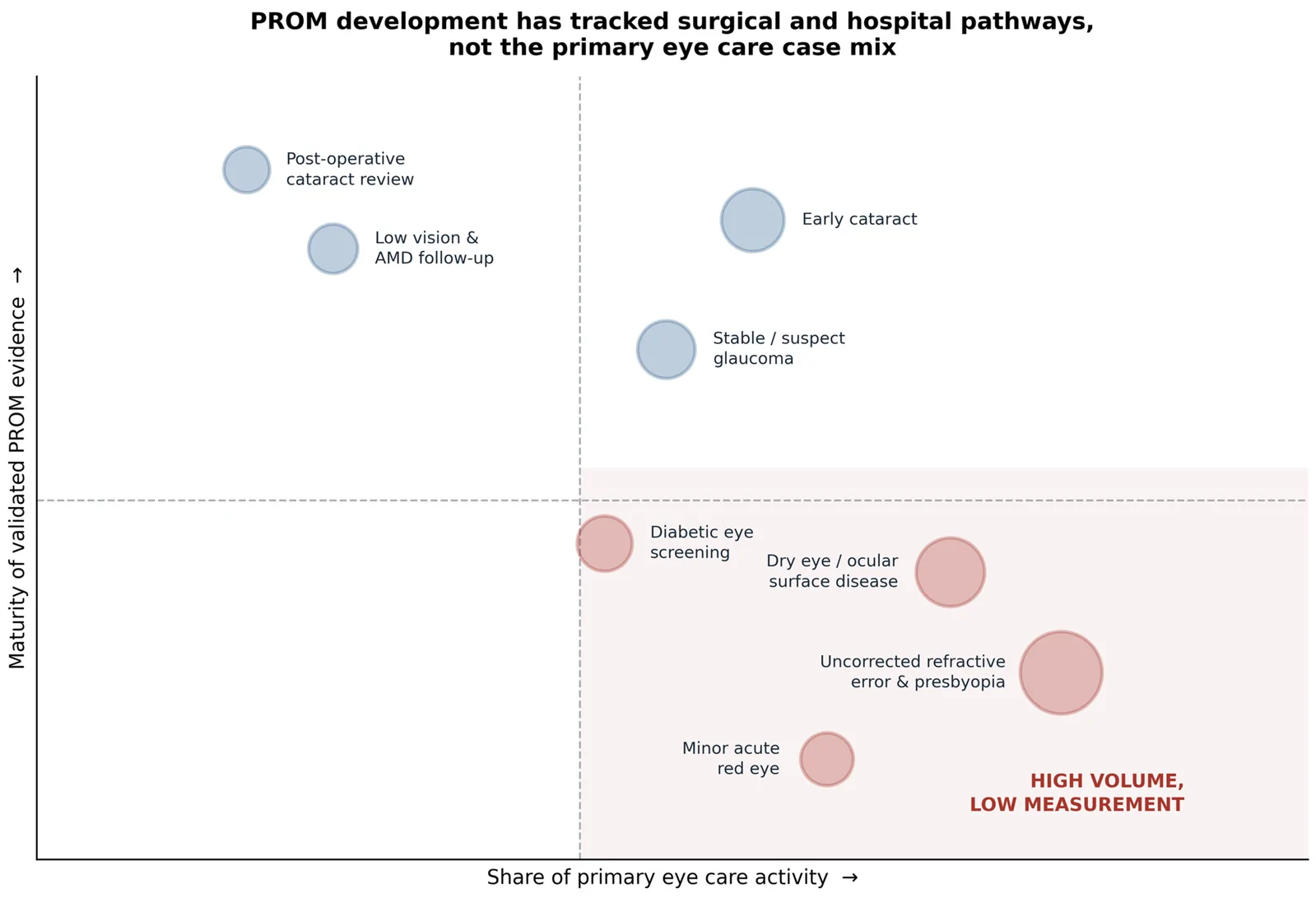
PROM development has followed surgical and hospital pathways rather than the primary eye care case mix. The shaded quadrant contains the highest-volume presentations in community practice and the least mature measurement evidence. This figure is a conceptual map, not a quantitative analysis: positions reflect the authors’ appraisal of the literature reviewed, no patient-level or survey data underlie it, and the axes carry no units.

Hospital ophthalmology, by contrast, concentrates advanced and surgical disease: cataract waiting lists, low-vision clinic referrals, established macular degeneration, and glaucoma with measurable field loss. The instruments discussed in Section 4 were built in that second population. Table 1 sets the two case mixes against the availability of validated PROM evidence, and Figure 2 maps the same relationship.

### 3.2. How the Framework Might Differ Across Health Systems

The definition above deliberately spans several health systems, but the evidence that we draw on is not evenly distributed across them, and that asymmetry affects how the framework should be read. The service-delivery and financing evidence in Section 6 and Section 7.3 is predominantly British, reflecting where community eye care has been most formally evaluated [9,20]. The access evidence in Section 5 comes from a single clinic in the United States [21]. The largest patient-reported effect sizes that we cite come from a low-resource setting in East Africa [11]. We therefore expect the five proposed gaps to differ in prominence between systems, and we set out that expectation here so that readers outside the United Kingdom can judge which parts of the argument transfer.

In publicly funded systems with commissioned community eye care, the targeting and action gaps are likely to dominate, because a delivery infrastructure already exists and the limiting factors are instrument performance and the absence of a defined route from score to decision. The funding gap takes the specific form of activity falling outside a fixed national fee, as the English that needs an assessment that we cite illustrates [20].

In insurance-based systems, the funding gap would be expected to take a different form: the constraint is less the absence of any payment route than the absence of a billable code or quality incentive attached to patient-reported measurement, while the access gap may be compounded by variation in coverage. The United States screening programme that we cite was designed around a safety-net population for related reasons [22].

In low- and middle-income settings, where primary eye care is often delivered by ophthalmic clinical officers or outreach teams, the evidence gap is likely to be the binding constraint, since few instruments have been developed or validated in these populations, and the access gap may be dominated by literacy and language rather than by digital exclusion. At the same time, the Zanzibar data indicate that interventions deliverable at this level produce the largest patient-reported gains in the literature that we reviewed [11], which makes the measurement deficit more, rather than less, consequential.

We have not identified evidence that would allow these systems to be ranked empirically, and we present this comparison as a structured expectation to be tested rather than as a finding.

## 4. The Targeting Gap

The instruments that dominate ophthalmic PROM practice were developed in populations with established, often advanced, disease. The NEI VFQ-25 was constructed from a cohort recruited across eleven university-based ophthalmology practices, comprising patients with age-related cataract, macular degeneration, diabetic retinopathy, primary open-angle glaucoma, cytomegalovirus retinitis, or low vision [2]. The VF-14 was derived from 766 patients undergoing the first cataract surgery [15]. The Impact of Vision Impairment questionnaire was evaluated in 314 first-time referrals to low-vision clinics [19]. Catquest-9SF was Rasch-scaled on 10,486 questionnaires drawn from the Swedish National Cataract Register and tested in a further 10,886, that is, on patients proceeding to surgery [14].

These are appropriate development populations for the purposes that those instruments were designed to serve. The difficulty arises when the same instruments are transplanted into a population sitting substantially lower on the underlying disability continuum.

### 4.1. What the Psychometric Evidence Shows

Rasch analysis makes targeting explicit: it places persons and items on a common logit scale, so that mismatch between item difficulty and person ability becomes measurable rather than assumed. Applied to the NEI VFQ, the results have been consistently unflattering. Pesudovs and colleagues found the NEI VFQ-39 and NEI VFQ-25 had good measurement precision but exhibited multidimensionality, misfitting items, suboptimum targeting of item difficulty to person ability, and dysfunctional subscales, twelve of them in the 25-item version [16]. Their conclusion was that the instrument is flawed as an overall measure and requires segregation into separate visual functioning and socioemotional scales. This was subsequently replicated in an independent Chinese cataract waiting-list cohort, where the original questionnaire was, in the authors’ words, once again found to be flawed [23].

Lundström and Pesudovs reported that items were well targeted to preoperative participants, with a 0.34 logit difference between person and item means [14]. But a systematic review of thirteen Catquest-9SF validation studies across diverse populations, totalling 15,289 respondents, found significant mistargeting in nine of them, with a range of 0.66 to −2.04 logits and, in most studies, the tasks being easy relative to respondent ability [24].

That last clause deserves emphasis. Mistargeting in the direction of items being too easy was observed in populations still sick enough to be on a cataract surgery waiting list. Primary eye care sees patients earlier and milder than that. We are not aware of evidence that targeting improves as severity falls, and the logic of the measurement model indicates that it should worsen; we therefore advance this as an inference rather than an observation, and Section 10 states the limits of that inference explicitly. No published Rasch analysis of a vision PROM in an unselected primary eye care population was identified.

### 4.2. Why This Matters Clinically

**Figure 3:**
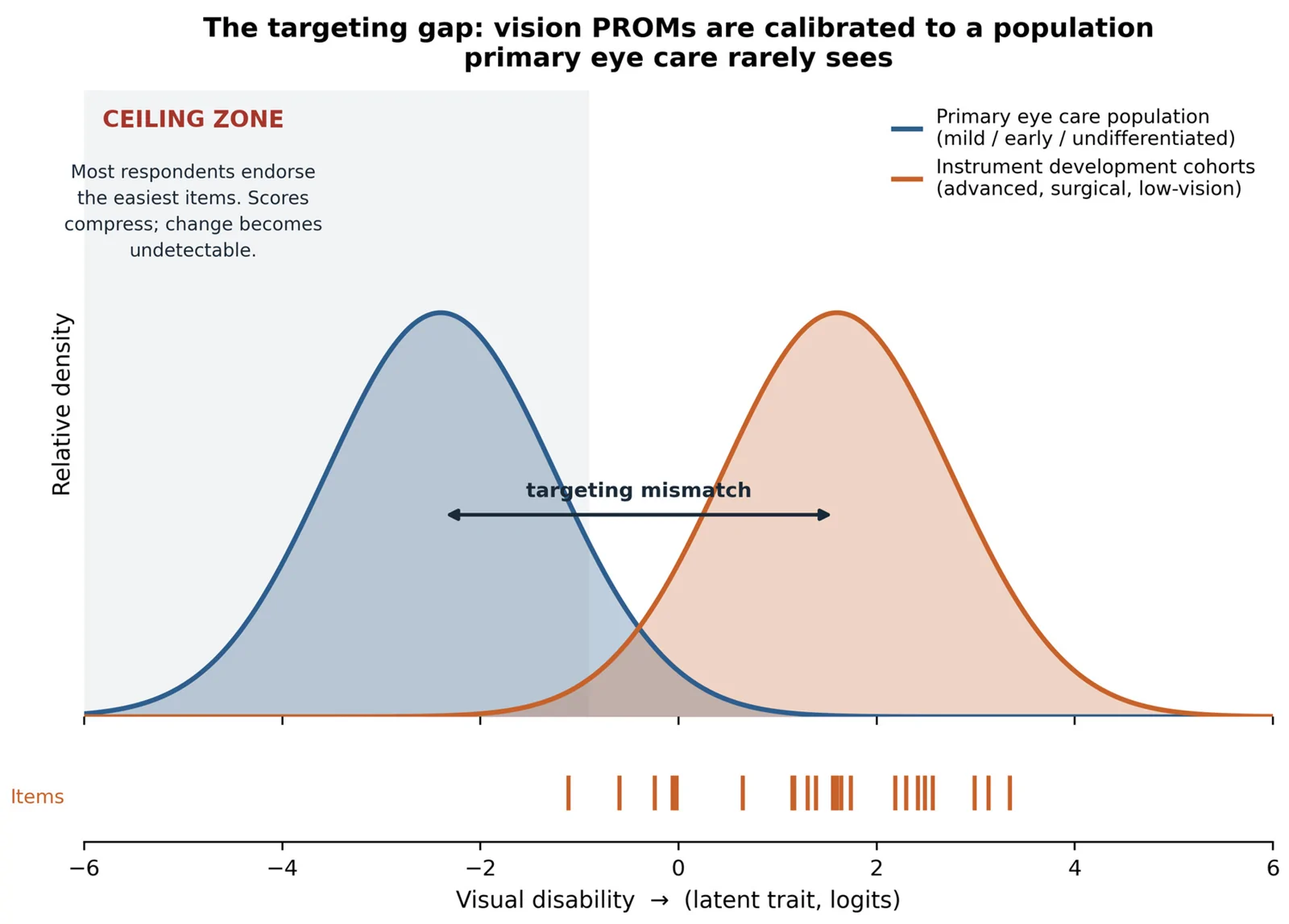
The targeting gap. Vision PROM items cluster at difficulty levels calibrated to hospital and surgical populations, while primary eye care patients sit lower on the disability continuum. The distributions shown are schematic illustrations of a measurement principle and are not derived from patient-level data, from any cited cohort, or from any statistical model; they are drawn to represent the direction of the mismatch documented in the Rasch literature cited [16,23,24], not to quantify it. The position of the primary eye care distribution is an extrapolation, since no Rasch analysis in such a population has been published.

Poor targeting is not an abstract psychometric complaint. It has three consequences that bear directly on whether a PROM can be useful in a community consultation (Figure 3).

- Ceiling effects. When most respondents can perform most of the tasks that an instrument asks about, scores compress at the favourable end of the scale. A patient with early cataract who reports no difficulty with any of nine daily activities is indistinguishable from a patient with no cataract at all.
- Insensitivity to change. Longitudinal monitoring depends on the instrument being able to register deterioration. If a patient begins at the ceiling, meaningful early decline is invisible until it becomes severe, which is precisely when the instrument is no longer needed to detect it.
- Inflated sample size requirements. Reduced measurement precision at the relevant end of the scale means that studies attempting to demonstrate a PROM-driven benefit in primary care need larger samples than equivalent hospital studies, making the evidence harder and more expensive to generate. This may partly explain why that evidence does not exist.

A related problem compounds this. Interpreting any PROM score requires knowing what magnitude of change is meaningful to a patient, yet minimal important differences in ophthalmic research remain inconsistently established [25]. Where estimates do exist, they are necessarily derived from the cohorts in which the instruments were developed, which, as set out above, are hospital and surgical populations; we record this as an inference from the development literature rather than as a separately evidenced finding. A clinician in a community setting therefore faces not only a score of uncertain precision but no reliable benchmark against which to judge it.

Braithwaite and colleagues reached a compatible conclusion in their review of PROM research in modern ophthalmology, noting considerable progress in cataract, glaucoma, medical retina, and low vision, alongside a paucity of useful tools for less common conditions, and proposing item banks administered by computer-adaptive testing as a route past the traditional limitations of fixed-form instruments [3]. Computer-adaptive testing is directly relevant here: by selecting items matched to each respondent’s estimated ability, it addresses targeting at the level of the individual rather than the cohort.

## 5. The Access Gap

The second gap has received remarkably little attention, and we consider it the most consequential for the interpretation of existing data.

The clearest evidence comes from the only published implementation of PROMs in a primary eye care setting that reports full uptake data. Over two months, general health and functioning PROMIS surveys were offered to all patients attending an optometric clinic in Rochester, New York. Of 651 patients, 258 chose to attempt the survey and 193 completed it, in a mean time of 6.36 min with a range extending from 1.43 to 51.92 min [21]. Overall completion was therefore 29.6% of those attending.

The critical finding is not the completion rate but its distribution. In this cohort, patients with low visual acuity were significantly less likely to attempt the survey (*p* = 0.049) [21]. Younger patients were more likely to complete it. No other patient characteristic distinguished participants from non-participants.

The implication, if this finding generalises, is uncomfortable. A self-administered instrument designed to quantify the impact of poor vision would systematically under-sample patients with poor vision. On this reading, the attrition would not be random but informative, structurally correlated with the construct being measured, and mean vision-related quality-of-life scores derived from self-administered collection in community settings would be optimistically biased by an unknown margin. We emphasise the conditional form of this claim. It is an inference from one single-centre study of a general-health instrument, with a *p*-value of 0.049, in one United States optometry clinic; it has not been replicated, and it has not been demonstrated for any vision-specific PROM. We therefore advance it as a hypothesis rather than as an established property of community PROM data. It is directly testable, and Section 9.1 sets out the reporting standard that would test it.

The wider implementation literature is consistent with this. A systematic review with qualitative evidence synthesis of electronic PROM collection in older adults identified clinical conditions, socio-cultural factors, digital literacy, access to digital technology, and user interface design as the factors most salient to older patients, with 29 of 34 review findings graded at high or moderate confidence [7]. An umbrella review of 34 reviews found no consensus favouring paper or electronic administration, but identified offering both options as an important facilitator specifically to mitigate digital literacy bias [6].

If this finding replicates, the practical conclusion is that assisted, large-print, audio, and interviewer-administered formats should be treated as the default design requirement for vision PROMs, not as an accommodation offered on request. An instrument that only the better-sighted can complete is not measuring what it claims to measure. We note that this recommendation follows from the hypothesis rather than from demonstrated bias and that it is, in any case, consistent with the independent evidence on digital exclusion among older adults cited above [6,7].

## 6. The Action Gap

Even where PROMs are collected in eye care, the data are rarely returned to the encounter that generated them.

This matters because feedback is the mechanism of benefit. The Cochrane review of PROM feedback identified 116 randomised trials across psychiatry, primary care, oncology, and other disciplines, involving 49,785 patients [4]. It found moderate-certainty evidence that feedback probably improves quality of life (SMD 0.15, 95% CI 0.05 to 0.26), increases patient–physician communication (SMD 0.36, 95% CI 0.21 to 0.52), improves diagnosis and notation (RR 1.73, 95% CI 1.44 to 2.08), and improves disease control (RR 1.25, 95% CI 1.10 to 1.41). A subsequent meta-analysis restricted to oncology found comparable improvements in health-related quality of life, communication, and one-year survival [26].

Notably, the effects concentrate in communication, diagnosis, and notation, outcomes that arise from a clinician seeing a score and responding to it. Collection alone does not produce them. This is the step missing in eye care.

Where PROMs and patient-reported experience measures have been deployed in community eye services, the data have been analysed in aggregate for a service evaluation. A community-based glaucoma clinic in Cambridge administered a modified glaucoma patient-reported outcome and experience measure to 96 consecutive patients, finding that 96% reported the experience as comfortable and 93% judged it equivalent to what they would expect from hospital care [27]. These are valuable findings about the acceptability of community delivery. They are not, and were not designed to be, instances of PROM data altering an individual patient’s management.

**Figure 4:**
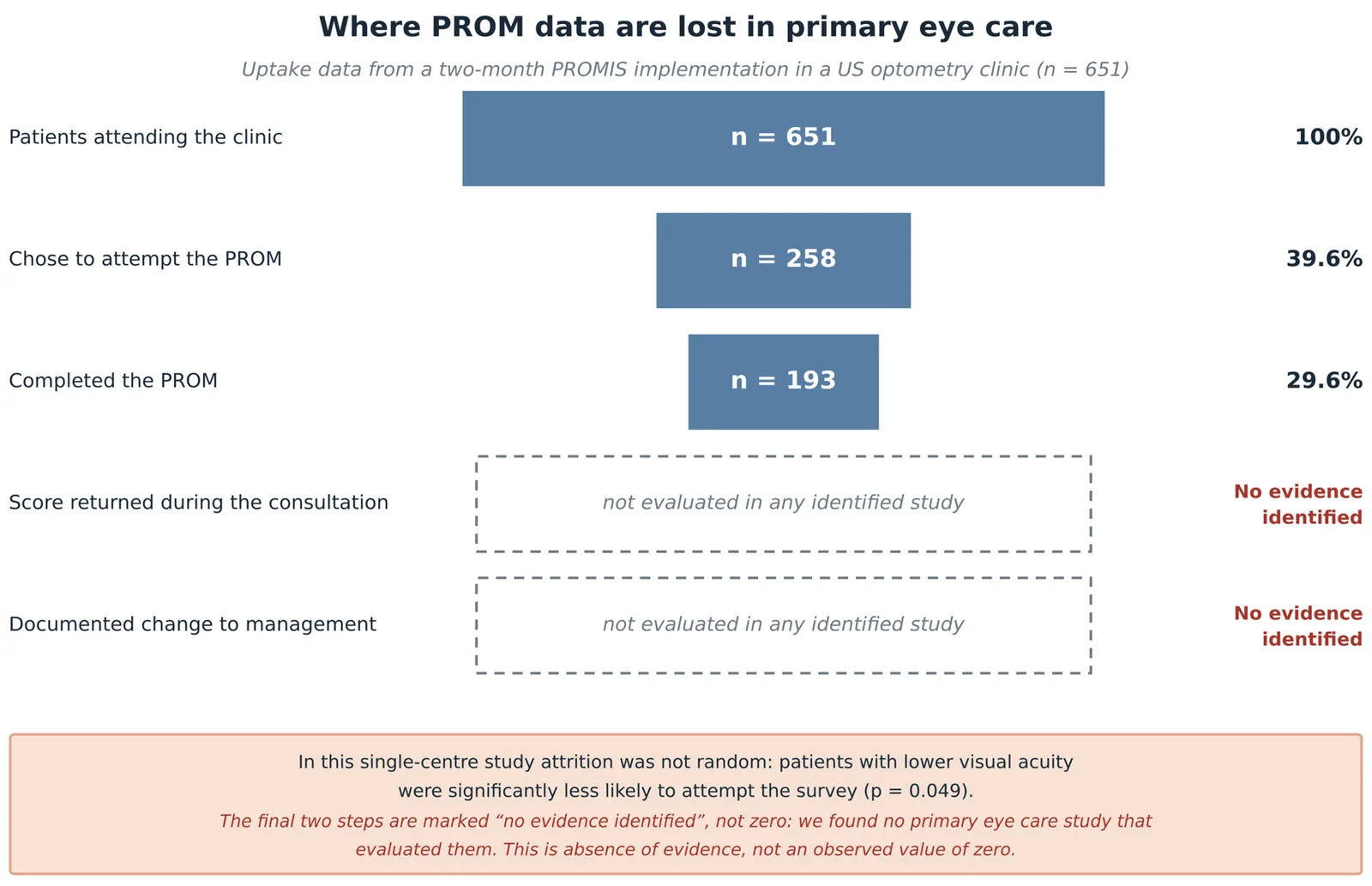
Attrition of PROM data in primary eye care, using uptake data from a two-month PROMIS implementation in a single US optometry clinic (n = 651) [21]. The first three stages are observed values from that study. The final two stages are labelled “no evidence identified” because no primary eye care study evaluating them was located; they are not observed values of zero, and the dashed outline is used to mark them as unevaluated rather than measured.

We could identify no published study in primary eye care demonstrating a PROM score returned to a clinician during a consultation and used to modify a decision. Figure 4 accordingly marks these two stages as “no evidence identified” rather than as zero, since they have not been evaluated in any study that we located. This is absence of evidence rather than evidence of absence, and the distinction matters: we are not reporting that such use was measured and found not to occur.

The European survey finding noted in the introduction bears repeating in this context: perceived usefulness did not predict adoption [5]. Clinicians are not failing to use PROMs because they doubt their value. They are failing to use them because the operational path from a completed questionnaire to a changed decision does not exist in most community services. Building conviction will not build that path.

Where a path has been sketched, it has been promising. A proposal to combine visual acuity with patient-reported outcome measures in an appropriateness and prioritisation tool for cataract surgery illustrates how patient-reported disability might reorder access on grounds other than acuity alone [28]. This remains a proposal requiring evaluation rather than established practice, but it is the right shape: a defined score, a defined threshold, and a defined consequence.

## 7. The Evidence Gap, and Where the Best Opportunity Lies

The fourth gap follows from the first three. Because instruments were built for hospital populations, because the patients most affected complete them least, and because collected data are not acted upon, there has been little incentive to generate evidence in the settings where most eye care now happens.

We suggest, however, that the field has been looking in the wrong place. The search for a global vision-related quality-of-life measure suitable for primary care has proved difficult for the psychometric reasons set out above. But there exists a category of patient-reported measurement that is brief, already validated, immediately actionable in a community consultation, and almost entirely neglected: measures of treatment administration and adherence.

### 7.1. Eye-Drop Technique and Adherence as the Model Case

A cross-sectional observational study in 136 Belgian community pharmacies assessed the eye-drop technique and patient-reported problems in 678 established eye-drop users with a mean age of 68.9 years [17]. Although 98.0% successfully instilled at least one drop, 14% required multiple attempts and only 3% performed every step correctly. Common deviations included touching the bottle to the eye or eyelid (40.7%), failing to close the eye afterwards (67.8%), and failing to perform nasolacrimal occlusion (94.7%). One in five ophthalmic suspensions was not shaken before use. Forty per cent of patients reported at least one problem with instillation, most often difficulty getting a drop into the eye (18.3%), too many drops emerging from the bottle (14.6%), or difficulty squeezing the bottle (12.2%). Roughly half recalled ever having received instruction in the technique.

Separately, patients whose adherence fell below 80% based on electronic monitoring and those scoring lower on a general glaucoma medication adherence self-efficacy scale were significantly more likely to have worse visual field defect severity [18].

As an important qualification, these studies establish that the eye-drop technique and adherence are common, consequential, and remediable problems. They do not, on their own, establish that the patient-reported measurement of those problems performs well, because both relied substantially on observations rather than self-report: the technique was assessed via demonstration in the pharmacy study [17] and via video recording with electronic monitoring of adherence in the glaucoma study [18]. The 40% figure for self-reported instillation problems is genuinely patient-reported; the 3% figure for perfect technique is observed. The gap between what patients report and what they do is precisely what is unknown here, and it matters: a self-reported technique measure that misses most of the errors that an observer would catch would be worse than useless. Validating brief self-reported technique and adherence items against observed performance in a community population is, in our view, the single most tractable and highest-yield study in this field.

Consider what this combination represents. A patient-reported measure that takes under two minutes, requires no visual acuity to complete, identifies a problem present in 40% of users, has an immediate and low-cost intervention available in any community setting, and is associated with the clinical outcome that matters. It satisfies every criterion that the vision-related quality-of-life instruments struggle to meet. It is also, as far as we can establish, almost never collected systematically in primary eye care.

### 7.2. Refractive Correction and the Equity Case

A second underexploited area is refractive and presbyopic correction. In a population-based survey in Zanzibar, presbyopia prevalence was 89.2% with spectacle coverage of only 17.6%; at six-month follow-up, 93.6% of those given spectacles still had them, mean satisfaction was 89.5%, and correcting presbyopia produced an effect size of 2.90 based on visual function and effect sizes ranging from 1.15 to 3.90 across quality-of-life domains [11]. These are among the largest patient-reported effect sizes in the ophthalmic literature, generated by an intervention deliverable at the primary level.

Community screening programmes offer a further route. The Michigan Screening and Intervention for Glaucoma and Eye Health Through Telemedicine programme was designed to deliver glaucoma screening in a federally qualified health centre and a free clinic, combining telemedicine review with health coaching and the provision of low- or no-cost spectacles, with vision-related quality of life among its stated outcomes [22]. Programmes of this kind place patient-reported measurement in exactly the setting where the access gap bites hardest, and their evaluation deserves close attention.

The equity dimension deserves emphasis. Low vision identified in general practice has been shown to carry poor function and inadequate support: in three West Glamorgan practices, the mean NEI VFQ score among registered blind and partially sighted patients was 41.5%, and although 80% had received a social services visit, only a third remained in contact [29]. The psychosocial burden of macular degeneration is comparable to that of major chronic systemic illness [30], and vision-related quality of life declines measurably with severity and the bilaterality of impairment, while generic instruments such as the SF-12 detect no difference at all [31]. Generic measures are insensitive here; vision-specific measures work, but only if the people with the worst vision can complete them, which returns us to the access gap.

### 7.3. The Funding Gap That Runs Beneath the Others

A further obstacle appears to cut across the other four and may be the most powerful of all, although the evidence available to us is indirect and almost entirely British: little of the activity that we have described appears to be funded.

A needs assessment conducted across Leeds, Bradford, and Airedale, areas without a commissioned Minor Eye Conditions Service, recorded how patients presenting to optometry with minor eye conditions were actually paid for. Optometrists managed 75% of patients. But 53% of patients paid privately for the appointment, 9% were absorbed into a General Ophthalmic Services sight test, 19% were seen without charge through practitioner goodwill, and 19% received no consultation at all and were redirected to a pharmacy, general practitioner, or emergency department. Some 34% would otherwise have attended an emergency department [20].

This is the economic context into which PROM collection is being proposed, at least in the United Kingdom. A community optometrist working to a fixed sight-test fee has, so far as we have been able to establish, no routine mechanism to bill for administering a questionnaire, for reviewing it, or for the additional consultation time that acting on it would require. We have not identified a published description of such a mechanism in any health system, but we note that the absence of a published description is weaker evidence than a documented absence, and that reimbursement arrangements may exist that are not reported in the indexed literature. Where enhanced activity has been commissioned and paid for, uptake has followed and outcomes have been demonstrable [9]. Where it has not, the work is either charged to the patient, absorbed as unpaid goodwill, or declined [20].

We suggest that this offers a more parsimonious explanation than clinician attitudes for the finding that perceived usefulness does not predict PROM adoption [5]. A practitioner may hold PROMs in high regard and still be unable to perform unfunded work at volume. If that is correct, then the substantial literature devoted to clinician education, awareness, and engagement has been addressing a genuine but secondary constraint. Figure 1 accordingly represents funding not as a fifth link in the chain but as the substrate on which all the others rest. We stress that this is a proposed explanation offered in competition with the attitudinal one and that the two have not been tested against each other.

Of the five proposed gaps, this is the one supported by the weakest and most geographically restricted evidence, and we advance it as a hypothesis rather than as a demonstrated mechanism. The reimbursement data that we cite describe minor eye condition consultations in three English areas, not PROM collection specifically, and no study has directly tested whether funding PROM activity increases it. That test is set out in Section 8.2. Any extension of this argument beyond the United Kingdom is an assumption on our part rather than a finding, and Section 3.2 sets out how we would expect the constraint to differ in insurance-based and low-resource systems.

## 8. The Case Against This Argument

A review that only marshals evidence for its own thesis is of limited use. We therefore set out the strongest position opposing ours, which we do not think is currently refutable.

### 8.1. Non-Adoption May Be Rational

The argument runs as follows. In a population with mild, early, or absent disease, a global vision-related quality-of-life score may genuinely carry very little decision-relevant information. If a patient with early cataract and 6/9 acuity reports no functional difficulty, the clinician has learned almost nothing that they did not already know from the history. On this reading, ceiling effects are not a defect of the instrument so much as an accurate reflection of a population that is, for the most part, functioning well. Clinicians may have arrived empirically at the conclusion that the measurement does not repay the time, and the failure of perceived usefulness to predict adoption [5] would then reflect a gap between abstract endorsement and situated judgement rather than a structural blockage.

This position has real force, and two observations support it. First, PROM feedback effects in the Cochrane synthesis, while positive, were modest for quality of life (SMD 0.15) and concentrated in communication, diagnosis, and notation [4], outcomes plausibly already achieved by a competent history in a low-complexity encounter. Second, most of the trials in that synthesis were conducted in populations with substantial symptom burden, where the prior probability of the questionnaire surfacing something unknown is far higher.

### 8.2. What Would Distinguish the Two Accounts

Our argument and the rational non-adoption argument make different predictions, and the distinction is empirically tractable.

- If non-adoption is rational, then instruments targeted to mild disease, through item banks or computer-adaptive testing, should still fail to change decisions, because the information deficit lies in the population rather than the instrument. If our account is right, better-targeted instruments should surface actionable findings at a materially higher rate.
- If non-adoption is rational, then eliminating the acuity-related response bias through assisted administration should not alter conclusions drawn from the data. If our account is right, assisted administration should shift observed mean scores downward and reveal unmet need concentrated in the previously missing respondents.
- If non-adoption is rational, funding PROM collection should not increase it much beyond a token level. If the funding gap is real, reimbursement should produce a step change, as it has for other enhanced community eye services [9,20].

None of these has been tested. We regard that as the most important finding of this review: the field has been debating whether clinicians should use PROMs without having established whether, in this population, they would learn anything.

### 8.3. Response Shift

A further complication applies to longitudinal use in chronic eye disease. Patients adapting to progressive impairment may alter their internal standards, values, and conceptualisation of quality of life over time, the phenomenon of response shift [32]. A patient whose visual function has deteriorated may report unchanged or even improved quality of life because the frame of reference has moved. In slowly progressive conditions such as glaucoma and macular degeneration, which are increasingly monitored in community settings, this undermines the interpretation of serial scores in exactly the pathway where longitudinal PROM monitoring is most often proposed. Symptom- and task-specific measures are less vulnerable to this than global quality-of-life instruments, which is a further argument for the sequencing that we propose below.

## 9. A Staged Route Forward

**Figure 5:**
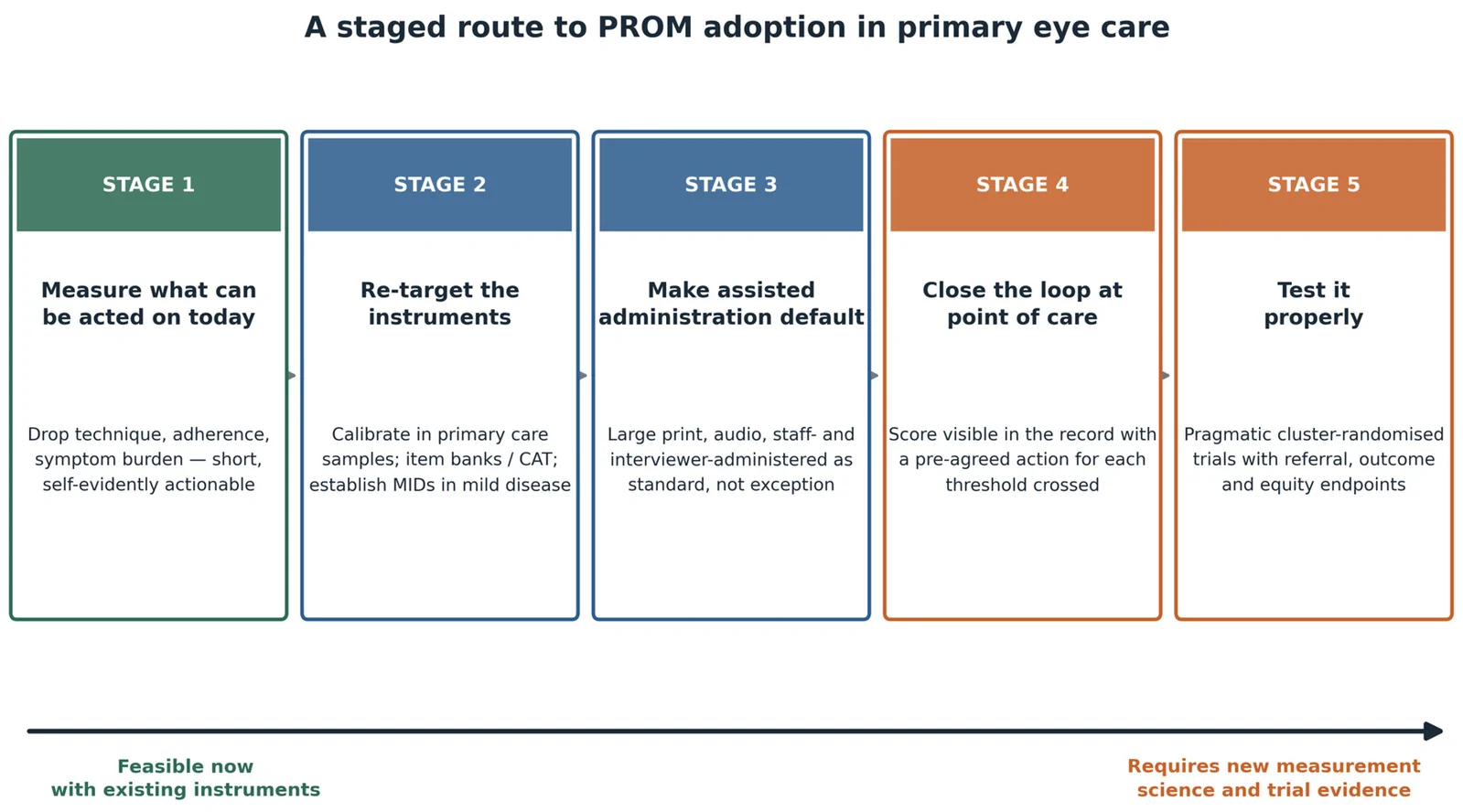
A staged route to PROM adoption in primary eye care, ordered by feasibility. Stages 1 and 3 are achievable with existing validated instruments; stages 2, 4, and 5 require new measurement development, service redesign, and trial evidence, respectively.

The five gaps are not equally tractable. Two can be addressed with instruments that already exist; three require new measurement science, service redesign, and trials. We therefore propose a sequence rather than a programme, on the principle that early demonstrable wins are what sustain implementation effort (Figure 5, Table 2).

### 9.1. Research Priorities

Several questions appear to us to be both answerable and consequential.

- Does routine collection of eye-drop technique and adherence measures in community pharmacy or optometry, with a defined remediation pathway, improve adherence and slow field loss in glaucoma? This is testable now with existing instruments in a cluster-randomised design.
- What are the psychometric properties of OSDI and DEQ-5 when administered in unselected primary eye care populations, and what are the minimal important differences in mild disease? Neither has been characterised in this setting.
- How large is the acuity-related response bias in self-administered vision PROMs, and does assisted administration eliminate it? This requires only that studies report attempt and completion rates stratified by visual acuity, a reporting standard that we would urge journals to adopt.
- Does returning a vision PROM score to a community optometrist before the consultation change referral decisions, and are those changes appropriate? The prioritisation literature suggests a mechanism [28]; no trial has tested it.
- Do brief self-reported eye-drop technique items agree with the observed technique in a community population? Existing evidence for technique failure is largely observational [17,18], and the validity of a self-reported substitute is unestablished.
- Does reimbursing PROM collection and the response within an enhanced service contract increase uptake, and by how much relative to education alone? This directly discriminates between the funding explanation and the attitudinal one.

### 9.2. A Worked Example of Closing the Loop

The instruction to define thresholds with pre-agreed actions is easy to state and rarely operationalised. Table 3 illustrates what it would mean concretely for a community glaucoma pathway, using only measures that already exist. Table 3 is an illustration of a design principle and must not be read as a clinical guideline or as a recommendation for immediate use. None of the thresholds that it contains has been validated, the instruments have not been tested together as a protocol, the intervals are conventional rather than evidence-derived, and the allocation of professional responsibility reflects United Kingdom community optometry practice and would need to be reassigned elsewhere. It is offered as a worked example of what closing the loop would require and as a template for local calibration and prospective evaluation.

Two design features distinguish this from conventional PROM collection. Every measure has a named action and a named person responsible, so that a score cannot be recorded without a consequence being specified. And no measure requires good visual acuity to complete, which addresses the access gap at the level of protocol design rather than as an afterthought. A third feature is the explicit separation of patient-reported screening from the observed assessment. The first row is a patient-reported screen whose function is to identify who needs their technique watched; the observation is the action triggered by the screen, not part of the measure itself. This distinction matters because, as Section 7.1 notes, the published evidence for technique failure is largely observational, and the agreement between what patients report and what they do has not been established.

## 10. Limitations

This review is purposive rather than systematic, and the risk of selective citation is real. We constructed an argument and then assembled evidence bearing on it; a differently constructed argument could be supported from the same literature. Readers should treat the five-gap framework as a hypothesis organising the available evidence rather than a finding established by it, and Section 8 sets out the principal competing account.

The targeting argument rests on extrapolation. This is the most serious weakness in our case, and we wish to state it plainly. The Rasch evidence that we rely on, namely Pesudovs and colleagues, its independent replication, and the Catquest-9SF synthesis [16,23,24], is derived entirely from hospital, cataract surgical, and low-vision cohorts. We are not aware of any published Rasch analysis of a vision PROM conducted in an unselected primary eye care population. Our inference that targeting deteriorates further as severity falls is grounded in the internal logic of the measurement model and in the observed direction of mistargeting, but it is an inference and not an observation. A systematic extraction of person and item mean locations across published Rasch analyses, plotted against the severity of each development population, would test it directly, and we regard this as the highest-priority methodological study arising from this review.

Our verification procedure relied on a single bibliographic database. PubMed does not index all relevant literature, particularly optometric, health services, and grey literature, and service evaluations of community eye care schemes frequently appear in reports rather than indexed journals. Restricting citation to verifiable indexed records improves accuracy at some cost to coverage and probably under-represents non-anglophone and low-income-country experience.

Our evidence base is also geographically uneven, and the framework should be read with that in mind. The service-delivery and financing evidence is almost entirely from the United Kingdom [9,20], the access evidence from a single clinic in the United States [21], and the largest patient-reported effect sizes from East Africa [11]. The five proposed gaps are therefore best supported in publicly funded systems with commissioned community eye care and least well supported in insurance-based and low-resource systems. Section 3.2 sets out how we would expect the framework to differ in those settings, but that section is an argued expectation rather than an evidenced finding, and we are not aware of comparative data that would test it.

Finally, the status of the five gaps should not be overstated. They are proposed explanations that organise the available evidence; they are not established causes, they have not been tested against one another, and their relative contribution is unknown. Each rests on evidence of differing strength, set out in Table 2, and the access and funding gaps, in particular, rest on single studies. Section 8.2 states the observations that would confirm or refute each of them.

Several claims rest on single studies. The access gap argument, in particular, derives its central evidence from one implementation study in one US optometry clinic [21]. That finding is biologically plausible, coheres with the wider literature on digital exclusion among older adults [7], and, to our knowledge, has not been contradicted, but it has also not been replicated, and a single *p*-value of 0.049 is a slender foundation for an argument of this weight. Replication should be a priority.

Observational designs predominate throughout the evidence base, with the attendant risks of confounding and selection bias, although the assumption that observational studies systematically overstate effects relative to randomised trials is itself not well supported [33]. Finally, we have proposed a staged implementation route that has not been tested as a whole; its components differ considerably in the strength of evidence behind them, as Table 2 indicates.

## 11. Conclusions

Patient-reported outcome measures have not reached primary eye care, and the standard explanation, that clinicians lack time, systems, and guidance, does not fit the evidence. Clinicians who consider PROMs useful are no more likely to use them than those who do not.

We have proposed five structural gaps that, taken together, may account better for the pattern and which we advance as hypotheses to be tested rather than as demonstrated causes. The instruments were calibrated on populations that primary eye care rarely sees and appear liable to mistarget when moved. Self-administration may systematically exclude the patients with the worst vision, in which case the data that are collected would be biased in a direction that flatters. Collected scores are banked for audit rather than returned to the consultation, which is the only step at which benefit has been demonstrated elsewhere. Measurement development has tracked surgical pathways, leaving the most common reasons for attending a community eye service essentially unmeasured. And beneath all of these, we identified no funding mechanism for the work, which may explain, more economically than any account based on clinician attitudes, why conviction and practice have come apart, although the evidence for this is British and indirect.

We have also set out the strongest argument against our own position: that in a largely well-functioning population, a vision quality-of-life score may simply carry little decision-relevant information and that clinicians have worked this out. We cannot currently refute this, and the studies that would discriminate between the two accounts have not been done. That, rather than any of our five gaps, may be the most useful thing that this review has to say.

None of this argues against PROMs in primary eye care. It argues against continuing to approach them as a problem of persuasion. The most promising route begins not with a better quality-of-life instrument but with short, already-validated, immediately actionable patient-reported constructs, whether a patient can get a drop into their eye, whether they are taking their treatment, and what their ocular surface symptoms are doing, collected in formats that do not require good vision to complete, returned to a clinician who has a pre-agreed action, and evaluated properly. That is a smaller ambition than a comprehensive vision PROM programme. It has the considerable advantage of being achievable now.

## Figures and Tables

**Table 1:** Primary eye care case mix against maturity of validated PROM evidence.

Presentation	Volume in Primary Eye Care	Candidate Instruments	State of the Evidence
Uncorrected refractive error and presbyopia	Very high	Near-vision and visual-function questionnaires; no consensus instrument	Large quality-of-life gains from simple correction are documented in low-resource settings, with effect sizes of 2.90 for visual function and 1.15–3.90 across quality-of-life domains [11]. No routine PROM use in high-income primary care.
Dry eye and ocular surface disease	High	OSDI (12 items) [12]; DEQ-5 (5 items) [13]	Both instruments are brief and validated and discriminate severity. Neither has been evaluated as a routine primary eye care measure.
Minor acute presentations (red eye, irritation)	High; the most common reason for attending community schemes, at 36.7% of presentations [9]	None established	No validated PROM developed for this population. The largest single measurement void in primary eye care.
Early cataract	Moderate	Catquest-9SF (9 items) [14]; VF-14 (14 items) [15]	Mature instruments, but calibrated on surgical cohorts; targeting deteriorates in milder disease [16].
Stable or suspect glaucoma under shared care	Moderate	Vision-specific and glaucoma-specific instruments; adherence and technique measures [17,18]	Adherence and eye-drop technique are patient-reported, actionable in primary care, and rarely collected.
Diabetic eye screening	Moderate	Generic and vision-specific instruments	Screening programmes measure attendance and yield, not patient-reported impact.
Low vision and macular degeneration follow-up	Low	NEI VFQ-25 [2]; IVI [19]	Well developed, but this is the population that primary care sees the least.

**Table 2:** The five proposed gaps, their evidential basis, the strength and provenance of that evidence, their consequences, and proposed responses. The third column is included so that readers can weigh each proposed gap against the evidence actually supporting it.

Gap	Evidential Basis	Strength and Provenance of Evidence	Consequence	Proposed Response
Targeting	Suboptimum item-to-person targeting and 12 dysfunctional subscales in the NEI VFQ-25 [16], replicated independently [23]; significant mistargeting in 9 of 13 Catquest-9SF validation studies, tasks easy relative to ability [24]	Moderate for the instruments as studied. Direct psychometric evidence, but derived entirely from hospital, cataract surgical and low-vision cohorts. Extension to unselected primary eye care populations is an inference; no Rasch analysis in such a population was identified.	Ceiling effects, insensitivity to early change, inflated sample sizes for primary care research	Calibrate instruments in primary care samples; develop item banks with computer-adaptive administration [3]; establish minimal important differences in mild disease [25]
Access	Patients with lower visual acuity significantly less likely to attempt a self-administered PROM (*p* = 0.049) [21]; digital literacy and interface identified as salient barriers for older adults [7]	Weak. One single-centre US study of a general-health instrument, not replicated, and not demonstrated for any vision-specific PROM. Advanced as a hypothesis requiring confirmation.	If confirmed, informative missingness correlated with the construct measured, and optimistically biased community vision-related quality-of-life means	Assisted, large-print, audio and interviewer-administered formats as default; offer paper and electronic in parallel [6]; report attempt and completion rates stratified by acuity
Action	PROM feedback improves communication, diagnosis and notation, and disease control across 116 trials [4], but no primary eye care study demonstrates point-of-care use; perceived usefulness does not predict adoption [5]	Moderate for the benefit of feedback, which rests on 116 randomised trials in other specialties [4]. The absence of point-of-care use in primary eye care is absence of evidence, not a measured null.	Data accumulate for an audit while consultations proceed unchanged; education-based remedies misdirected	Define score thresholds with pre-agreed clinical actions; surface scores in the record before the consultation; build on prioritisation models combining acuity and PROM data [28]
Evidence	PROM development concentrated in cataract, glaucoma, retina, and low vision [3]; red eye alone accounts for 36.7% of community scheme presentations with no validated instrument [9]	Moderate. Case-mix data derive from UK community schemes [9] and may not generalise to other systems; the absence of validated instruments for these presentations is a documented gap rather than an inference.	The most common reasons for attending are the least measured	Start with validated, actionable measures already suited to community use, namely eye-drop technique and adherence [17,18] and ocular surface symptoms [12,13]; evaluate with pragmatic cluster-randomised designs
Funding (cross-cutting)	53% of minor eye condition consultations paid privately, 19% absorbed as practitioner goodwill, 19% declined outright [20]; commissioned schemes achieve uptake and measurable outcomes [9]	Weak and geographically narrow. One needs an assessment in three English areas [20]; no study has tested reimbursement as an implementation lever. The weakest evidenced of the five proposed gaps.	Unfunded measurement may not be performed, regardless of clinician conviction; offered as a competing explanation for why perceived usefulness does not predict adoption [5]	Commission PROM collection and response as a specified, reimbursed component of enhanced service contracts; test reimbursement directly as an implementation lever

**Table 3:** Illustrative point-of-care protocol for a community glaucoma pathway. This table is a worked illustration of a design principle, not a validated clinical guideline and not a recommendation for use. No threshold shown has been clinically validated, the measures have not been evaluated together as a protocol, and the allocation of responsibility reflects United Kingdom community optometry practice. The second column distinguishes measures completed by the patient from the observed assessment that a positive patient-reported screen is intended to trigger.

Measure	Measurement Mode	Timing	Threshold Triggering Action	Action	Responsible
Self-reported eye-drop instillation problems (single screening item plus brief checklist)	Patient-reported screen; a positive screen triggers observed assessment	Every review visit	Any reported problem, or patient unable to describe technique	Positive screen prompts direct observation of instillation technique by the clinician; corrective instruction; consider compliance aid or alternative bottle	Optometrist or dispensing optician
Adherence self-efficacy (brief scale)	Patient-reported	Annually, and at any treatment change	Score in lowest tertile for the service	Structured discussion of barriers; simplify regimen where possible; flag to prescriber	Optometrist
Ocular surface symptoms (DEQ-5)	Patient-reported	At review, and where drops recently started or changed	Score above the published screening threshold	Review preservative exposure; consider preservative-free substitution; treat surface disease	Optometrist, with prescriber
Patient-reported change in visual function since last visit (single global item)	Patient-reported	Every review visit	Any reported deterioration, irrespective of measured acuity	Prompt formal reassessment; do not defer on the basis of stable acuity alone	Optometrist
Understanding of diagnosis (single item)	Patient-reported	At first visit and annually	Patient unable to state diagnosis or its implications	Structured education; written information; consider longer follow-up appointment	Optometrist

## Data Availability

No new data were created or analysed in this study. All data discussed are contained within the cited publications.

## Appendix A

The four domain searches below were run in MEDLINE via PubMed up to June 2026, with no language limits applied at the search stage. Selection from the retrieved records was purposive and is described in Section 2.2. Additional sources were identified via backward and forward citation tracking.

**Table A1:** PubMed search strings, by domain.

Domain	PubMed Search String
(i) Vision PROM development and psychometrics	(“patient reported outcome measures”[MeSH Terms] OR “patient-reported outcome”[Title/Abstract] OR “PROM”[Title/Abstract]) AND (“ophthalmology”[MeSH Terms] OR “vision”[Title/Abstract] OR “visual function”[Title/Abstract] OR “eye”[Title/Abstract]) AND (“psychometrics”[MeSH Terms] OR “Rasch”[Title/Abstract] OR “validation”[Title/Abstract] OR “targeting”[Title/Abstract])
(ii) PROM implementation in routine care	(“patient reported outcome measures”[MeSH Terms] OR “patient-reported outcome”[Title/Abstract]) AND (“implementation”[Title/Abstract] OR “routine clinical practice”[Title/Abstract] OR “barriers”[Title/Abstract] OR “facilitators”[Title/Abstract]) AND (“review”[Publication Type] OR “systematic review”[Publication Type] OR “meta-analysis”[Publication Type])
(iii) Community and primary eye care service evaluation	(“optometry”[MeSH Terms] OR “community optometry”[Title/Abstract] OR “primary eye care”[Title/Abstract] OR “minor eye conditions”[Title/Abstract] OR “shared care”[Title/Abstract]) AND (“service evaluation”[Title/Abstract] OR “referral”[Title/Abstract] OR “pathway”[Title/Abstract] OR “commissioning”[Title/Abstract] OR “patient satisfaction”[MeSH Terms])
(iv) Primary eye care case mix and epidemiology	(“eye diseases”[MeSH Terms] OR “dry eye syndromes”[MeSH Terms] OR “presbyopia”[MeSH Terms] OR “refractive errors”[MeSH Terms] OR “vision disorders”[MeSH Terms]) AND (“prevalence”[Title/Abstract] OR “epidemiology”[MeSH Subheading] OR “case mix”[Title/Abstract] OR “global burden”[Title/Abstract]) AND (“primary health care”[MeSH Terms] OR “community”[Title/Abstract] OR “population-based”[Title/Abstract])
